# Low‐Dose Interleukin‐2 in Patients with Mild to Moderate Alzheimer's Disease: A Randomized Clinical Trial

**DOI:** 10.1002/alz70859_101215

**Published:** 2025-12-25

**Authors:** Alireza Faridar, Nazaret Gamez, Michael Grundman, Belen Pascual, Joseph C. Masdeu, Stanley H Appel

**Affiliations:** ^1^ Houston Methodist Research Institute, Houston, TX USA; ^2^ Global R&D Partners, LLC, La Jolla, CA USA

## Abstract

**Background:**

We previously documented that regulatory T cells (Tregs) immunomodulatory mechanisms are compromised in Alzheimer’s disease (AD), shifting the immune system toward a pro‐inflammatory response. However, Tregs are a potentially restorable therapeutic target in AD. In this study, we evaluated the safety and efficacy of two dosing frequencies of low‐dose Interleukin‐2 (IL‐2) in expanding Regulatory T cells (Tregs) to modify disease progression in Alzheimer’s Disease (AD) patients.

**Method:**

In this phase 2a, randomized, double‐blind, placebo‐controlled study, 38 participants were assigned to receive subcutaneous IL‐2 (10^6 IU/day) for five days, administered either every 4 weeks (IL‐2q4wks) or every 2 weeks (IL‐2q2wks), versus placebo, for 21 weeks, followed by 9 weeks of observation. The primary endpoints were the incidence and severity of adverse events. For the secondary endpoints, changes in Treg numbers and suppressive functions were evaluated. Exploratory endpoints included changes in blood inflammatory mediators, cerebrospinal fluid (CSF) AD‐related biomarkers, and clinical scales.

**Result:**

Of the 38 participants, 9 received IL‐2q4wks, 10 received IL‐2q2wks, and 19 received placebo. All participants completed the trial with no serious adverse events or deaths. Both IL‐2 dosing regimens increased Treg numbers and suppressive function, but IL‐2q4wks treatment exhibited superiority in enhancing Treg percentage and Foxp3 mean fluorescent intensity. Consistently, in the longitudinal analysis of 45 inflammatory mediators, IL‐2q4wks administration demonstrated greater efficacy in suppressing the inflammatory mediators CCL2, CCL11, and IL‐15, while enhancing plasma anti‐inflammatory IL‐4 levels. A significant improvement in CSF Aβ42 levels (*p* = 0.045 vs. placebo) was observed following IL‐2q4wks administration. While CSF NFL increased by 217 pg/ml in placebo recipients, it remained stable in the IL‐2 q4wks group (p=0.060, IL‐2 q4wks vs. placebo). The adjusted mean change from baseline in the ADAS‐cog14 score at week 22 indicated a trend toward slower clinical progression in IL‐2q4wks recipients compared to placebo (p=0.061).

**Conclusion:**

The IL‐2 immunotherapeutic strategy was safe and well‐tolerated. IL‐2 q4wks effectively restored Treg populations, leading to modification in inflammatory mediators and AD biomarkers, while also showing promising trends in clinical scales. These findings provide a foundation for further investigation of low‐dose IL‐2 as a potential treatment for Alzheimer's Disease.

